# Wavelet Neural Network Using Multiple Wavelet Functions in Target Threat Assessment

**DOI:** 10.1155/2013/632437

**Published:** 2013-02-20

**Authors:** Gaige Wang, Lihong Guo, Hong Duan

**Affiliations:** ^1^Changchun Institute of Optics, Fine Mechanics and Physics, Chinese Academy of Sciences, Changchun 130033, China; ^2^Graduate School of Chinese Academy of Sciences, Beijing 100039, China; ^3^School of Computer Science and Information Technology, Northeast Normal University, Changchun 130117, China

## Abstract

Target threat assessment is a key issue in the collaborative attack. To improve the accuracy and usefulness of target threat assessment in the aerial combat, we propose a variant of wavelet neural networks, MWFWNN network, to solve threat assessment. How to select the appropriate wavelet function is difficult when constructing wavelet neural network. This paper proposes a wavelet mother function selection algorithm with minimum mean squared error and then constructs MWFWNN network using the above algorithm. Firstly, it needs to establish wavelet function library; secondly, wavelet neural network is constructed with each wavelet mother function in the library and wavelet function parameters and the network weights are updated according to the relevant modifying formula. The constructed wavelet neural network is detected with training set, and then optimal wavelet function with minimum mean squared error is chosen to build MWFWNN network. Experimental results show that the mean squared error is 1.23 × 10^−3^, which is better than WNN, BP, and PSO_SVM. Target threat assessment model based on the MWFWNN has a good predictive ability, so it can quickly and accurately complete target threat assessment.

## 1. Introduction

With the development of science and technology, the requirement of information is increasingly improving in modern warfare. To adapt to this change, many countries have begun the research of multisensor information fusion from the 1970s. After years of research, the United States, Britain, and other military powers have developed a number of information fusion systems which can be used for combat. Target threat assessment belongs to the third level in information fusion model and is a kind of high-level information fusion. The target threat assessment is the essential basis for the allocation of force and fire in C4ISR system.

The traditional methods to solve threat assessment are Bayesian inference [1, 2], multiattribute decision-making theory [3], GSOBP [4], Elman_AdaBoost [5], analytic hierarchy process [6], Dempster-Shafer theory [7], Hypothesis-drive [8], and so forth. These methods are based on constant weight vector and must rely on expertise available, which makes these methods significantly increase subjective factor of threat assessment, and it is highly possible to evaluate inaccurate results, making the complex relationship between evaluation indicators not effectively reflected. In addition, the models created by these methods have a fatal drawback; that is, these models do not have the self-learning and adaptive capacity, so it is difficult to adapt to change as the change of the enemy air attack weapons performance and tactical means brought to the weights of each factor changes. As the neural network has many advantages, such as strong learning ability and adaptability, it is adept at working out the target threat assessment compared with the above-mentioned methods. BP is a mature and effective method, with the advantages of rigorous derivation process, solid theoretical basis, strong versatility, and clear physical concepts. Literature [7, 8] studies threat assessment using BP networks, and achieve good results. However, with the dimension of training data increasing, the convergence of BP slows down, and the network performance deteriorates; moreover, in the training process, it is easy to fall into local minimum solution. Wavelet neural network (WNN) has many advantages compared with other neural networks; for example, the parameters (hidden nodes and weight) are more easily determined than the radial basis function (RBF) neural networks; it requires smaller training amount than multilayer perceptron network; also, wavelet neural network has a fast convergence. In the same approximation quality, the wavelet neural network requires fewer nodes. For WNN, one of the biggest drawbacks is the difficulty of the choice of mother wavelet function, so this paper proposes an algorithm for selecting optimal mother wavelet function. And then, we construct the (Multiple Wavelet Function Wavelet Neural Networks) MWFWNN using the above method to select optimal mother wavelet function for threat assessment.

## 2. MWFWNN Network

### 2.1. Wavelet Theory

Firstly proposed by Grossman and Morlet in the 1980s, wavelet theory [9] is a mathematical theory and analysis method to make up the shortages of Fourier transform. In the field of signal processing, the most widely used analysis method is the Fourier transform, but it has obvious deficiency that the Fourier transform has no distinguishable ability in the time domain, because the time information is not included in the results of Fourier transform. Wavelet is special waveform with the mean 0 and the limited length.

Wavelet function is constructed through a series of basic transformation with a mother wavelet function. Not all functions can be used as wavelet mother function if a wavelet function is to be available and then develop into a good wavelet transform function, it must satisfy many conditions. Therefore, it is difficult to find the practical wavelet function. In the practical wavelet functions, some of them do not have expressions.

Let *φ*(*t*) be a square integrable function, that is, *φ*(*t*) ∈ *L* ^2^(*R*). If its Fourier transform Ψ(*ω*) can satisfy the following compatibility condition:

(1) $$\begin{array}{c} \int_{R}^{}\frac{{\left|\Psi \left(\omega \right)\right|}^{2}}{\omega }\text{d}\omega <\infty \end{array}$$

then *φ*(*t*) is called a basic wavelet or mother wavelet function. We make translation and scale for wavelet function, the translation factor *τ*, and the scale factor (also known as the expansion factor) *a*, so that we get function *φ*_*a*,*τ*_(*t*):

(2) $$\begin{array}{c} \varphi_{a,\tau }\left(t\right)=a^{1/2}\Psi \left(\frac{t-\tau }{a}\right),\;a>0,\;\;\tau \in R. \end{array}$$

As the translation factor *τ* and the scale factor *a* are continuous variables, their value can be positive or negative; so Ψ_*a*,*τ*_(*ω*) is called continuous wavelet function (also called the mother wavelet function).

Wavelet transform calculates the inner product between the signal *x*(*t*) with mother wavelet function

(3) $$\begin{array}{c} f_{x}\left(a,\tau \right)=\frac{1}{\sqrt{a}}\int_{-\infty }^{\infty }x\left(t\right)\varphi^{*}\left(\frac{t-\tau }{a}\right)\text{d}t. \end{array}$$

Equivalent expression in time domain is given as

(4) $$\begin{array}{c} f_{x}\left(a,\tau \right)=\frac{\sqrt{a}}{2\pi }\int_{-\infty }^{\infty }X\left(\omega \right)\Psi^{*}\left(a\omega \right)e^{j\omega \tau }\text{d}\omega , \end{array}$$

where *a* > 0, *τ* ∈ *R*, *X*(*ω*) and Ψ(*ω*) are the Fourier transform of *x*(*t*) and *φ*(*t*), respectively.

The conclusion can be drawn from (3) and (4) that is wavelet analysis can analyze the local characteristics of the signal through the mother wavelet function transformation; therefore, wavelet theory is considered to be the breakthrough for the Fourier transform, and the theory has been successfully applied to image processing, optical devices detection, and signal analysis and other fields.

### 2.2. Wavelet Neural Network

Wavelet transform has time-frequency localization property and focal features and neural network (NN) has self-adaptive, fault tolerance, robustness, and strong inference ability. How to combine the advantages of wavelet transform and NN to solve practical problems has been one of the hot spots. So-called wavelet neural network (WNN) or wavelet network (WN) is a variety of two techniques and inherits the advantages of the neural network and wavelet transformation. Proposed by Q. Zhang in 1992 [10], WNN uses the wavelet function as the activation function instead of the Sigmoid activation function.

**Figure 1:**
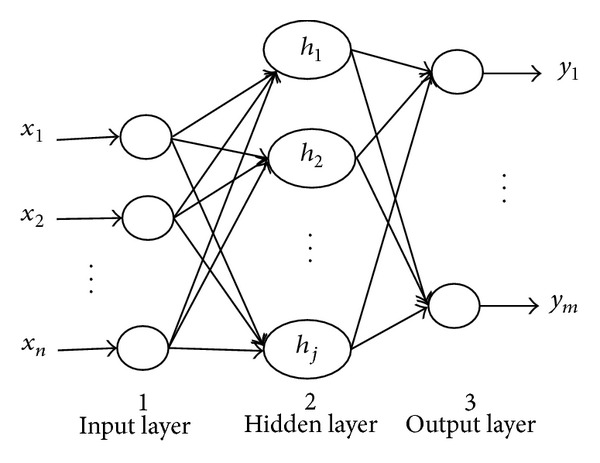
Topology of wavelet neural network.

For WNN, its topology is based on BP network; the transfer function of hidden layer nodes is the mother wavelet function; and the network signal is prior to transmission while error is backpropagation in the training process. The network topology is shown in Figure 1. In Figure 1, *x*_1_, *x*_2_,…, *x*_*n*_ is the input vector; *y*_1_, *y*_2_,…, *y*_*l*_ is the predicted output; *ω*_*ij*_ and *ω*_*kj*_ are the weights connecting every layer; and *h*_*j*_ is mother wavelet function.

For the input signal sequence *x* = (*x*_1_, *x*_2_,…, *x*_*n*_), the output of the hidden layer is calculated as

(5) $$\begin{array}{c} h\left(j\right)=h_{j}\left[\frac{\sum_{i=1}^{n}\omega_{ij}x_{i}-b_{j}}{a_{j}}\right],\;j=1,2,\ldots ,m, \end{array}$$

where *h*(*j*) is output value for the node *j* in the hidden layer; *h*_*j*_ is the mother wavelet function; *ω*_*ij*_ is weight connecting the input layer and hidden layer; *b*_*j*_ is the shift factor, and *a*_*j*_ is the stretch factor for *h*_*j*_.

Currently, the choice of mother wavelet functions has not yet formed a standard theory; commonly used wavelet functions are Morlet, Haar, Daubechies (dbN), Symlet (symN), Meryer, Coiflet, Biorthogonal wavelets, and so on.

The output of the output layer is calculated as

(6) $$\begin{array}{c} y\left(k\right)=\sum_{i=1}^{m}\omega_{ik}h\left(i\right),\;k=1,2,\ldots ,l, \end{array}$$

where *h*(*i*) is the output value for node *i* in the hidden layer; *ω*_*ik*_ is weight connecting the hidden layer and output layer; *l* and *m* are the number of nodes for output layer and the hidden layer, respectively.

For WNN, the updating weight algorithm is similar to BP network; the gradient method is used to update mother wavelet function parameters and connection weights between the layers, making the prediction output closer and closer to the desired output. The weights of WNN and the parameters of wavelet function are updated as follows.

(1) Calculating the prediction error of WNN

(7) $$\begin{array}{c} e=\sum_{k=1}^{m}yn\left(k\right)-y\left(k\right), \end{array}$$

where, *y*(*k*) is the predicted output value, *yn*(*k*) is the expected output value for the network.

(2) Updating the weights of WNN and the parameters of wavelet function according to the prediction error *e*

(8) $$\begin{array}{c} \omega_{n,k}^{\left(i+1\right)}=\omega_{n,k}^{\left(i\right)}+\Delta \omega_{n,k}^{\left(i+1\right)},\\ a_{k}^{\left(i+1\right)}=a_{k}^{\left(i\right)}+\Delta a_{k}^{\left(i+1\right)},\\ b_{k}^{\left(i+1\right)}=b_{k}^{\left(i\right)}+\Delta b_{k}^{\left(i+1\right)}, \end{array}$$

where Δ*ω*_*n*,*k*_^(*i*+1)^, Δ*a*_*k*_^(*i*+1)^, and Δ*b*_*k*_^(*i*+1)^ are calculated by the network prediction error:

(9) $$\begin{array}{c} \Delta \omega_{n,k}^{\left(i+1\right)}=-\eta \frac{\partial e}{\partial \omega_{n,k}^{\left(i\right)}},\\ \Delta a_{k}^{\left(i+1\right)}=-\eta \frac{\partial e}{\partial a_{k}^{\left(i\right)}},\\ \Delta b_{k}^{\left(i+1\right)}=-\eta \frac{\partial e}{\partial b_{k}^{\left(i\right)}}, \end{array}$$

where *η* is the learning rate.

The process of training WNN is as follows

1. Data preprocessing: first, the original data is quantified and normalized, and then the data is divided into training set and testing set for network training and testing, respectively.
2. Initializing WNN: connection weights *ω*_*ij*_ and *ω*_*jk*_, translation factor *b*_*k*_, and scale factor *a*_*k*_ are randomly initialized, and the learning rate *η* is set.
3. Training network: input the training set into WNN, compute network predicted output values, and calculate the error *e* between output and the expected value.
4. Updating the weights: update mother wavelet function parameters and network weights according to the prediction error *e*, making the predictive value of the network as close to actual values.
5. If the results satisfy the given conditions, use the testing set to test the network, otherwise, return to Step 3.

### 2.3. MWFWNN

MWFWNN will be provided in this section.

#### 2.3.1. MWFWNN Algorithm

**Step 1**

Initializing: initialize mother wavelet function library waveFunction = {waveFunction_*i*_},  *i* = 1,2,…, *K*, *K* = ||waveFunction | | is the number of elements waveFunction included, the variance *δ*_*i*_ for mother wavelet functions waveFunction_*i*_, and optimal wavelet function waveFunction_best_ =  waveFunction_1_ and its variance *δ*_best_ = *δ*_1_.

**Step 2**

Choosing the best mother wavelet function: for each mother wavelet function,

(i) Update the weights and parameters of wavelet function waveFunction_*i*_ according to (7)–(9).
(ii) if *δ*_*i*_ < *δ*_best_
  (10) δbest=δiwaveFunctionbest=waveFunctioni
  End

**Step 3**

Constructing MWFWNN using waveFunction_best_ as mother wavelet function.

**Step 4**

Testing constructed MWFWNN network in Step 3 using the testing set.

**Step 5**

Analyzing results.

## 3. Target Threat Assessment Using MWFWNN

Strictly speaking, threat assessment is an NP-hard problem, belonging to the third level in the JDL information fusion model. Target threat assessment needs to consider many factors (such as geography, weather, enemy, etc.), and the relation among the various factors is not a simple linear combination and it is difficult to determine a function between the target threat value and various factors. Therefore, we must consider various factors and their relationships when studying the threat assessment. However, we consider the following six factors in general: target type, target speed, target heading angle, target height, and target distance. We will test the performance of MWFWNN using these factors in this paper.

### 3.1. Target Threat Assessment Factor

We mainly consider the following six key factors when studying the target threat assessment in the paper:

1. Target Type: large targets (such as fighter-bombers), small targets (such as stealth aircraft, cruise missiles), and helicopters;
2. Target heading angle: such as 22°, 26°, and 6°;
3. Target speed: such as 100 m/s, 500 m/s, and 220 m/s;
4. Target height: such as very low, low, medium, and high;
5. Target interference: such as strong, medium, and weak;
6. Target distance: such as 100 km, 110 km, and 220 km.

### 3.2. Threat Assessment Model Using MWFWNN

**Figure 2:**
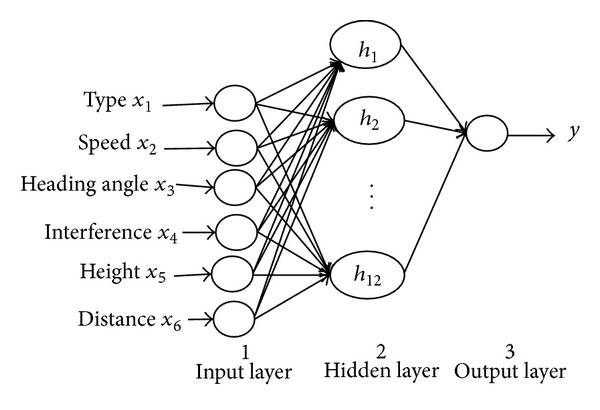
Architecture for the model of target threat assessment based on MWFWNN.

We design MWFWNN model according to the data characteristics. Because the data is 6-dimensional, and the output is 1-dimensional, the structure of WNN is 6-12-1. Firstly, we input six indicators that are the target type, target speed, target heading angle, target interference, target height, and the distance to the input layer. The hidden layer nodes are formed by the wavelet function, and the output layer outputs predicted target threat assessment value under the current indicators. On the basis of the above analysis, we construct the target threat assessment model based on MWFWNN with these six selected indicators, and its architecture is shown in Figure 2.

## 4. Model Simulation

In this section, we will test the target threat assessment model using MWFWNN proposed in Section 3.

### 4.1. Data Preprocessing

Part of the data used in our work is shown in Table 1. Target value is quantified using  G. A. Miller's quantitative theory, which represents the degree of threat: extremely small, very small, little small, small, medium, large, little large, very large, and extremely large. The properties of the specific quantitative criteria are quantified as follows.

1. Target Type: helicopter, large target (such as fighter-bombers), and small targets (such as stealth aircraft, cruise missiles), are quantified by 3, 5, and 8, respectively;
2. Target interference: strong, medium, weak, and no are quantified by 8, 6, 4, and 2, respectively;
3. Target height: very low, low, medium, and high are quantified 8, 6, 4, and 2, respectively;
4. Target speed: 0 m/s~1800 m/s equal interval (200 m/s) is quantified by 9 to 1.
5. Target heading angle: 0° ~ 36° equal interval (4°) is quantified by 9 to 1.
6. Target distance: 0 km~450 km equal interval (50 km) is quantified by 9 to 1.
7. Determining the target output: firstly normalize the various factors from air combat situation and then put them into the WMF_WNN proposed in Section 3.2. At last, the WMF WNN outputs threatening assessment value.

After quantifying the data, we can normalize the training set and testing set using the following expression:

(11) $$\begin{array}{c} f:x\to y=\frac{x-x_{\min}}{x_{\max}-x_{\min}}, \end{array}$$

where *x*, *y* ∈ *R* ^*n*^, *x*_min⁡_ = min⁡(*x*), *x*_max⁡_ = max⁡(*x*). The values are converted into the range [0, 1] through the normalization, that is, *x*_max⁡_ ∈ [0,1], *i* = 1,2,…, *n*.

### 4.2. Analysis of Simulation Results

In this paper, we implement the MWFWNN algorithm by MATLAB R2009a with the CPU Pentium (R) 4 3.06 GHz, 1 G memory (2∗512 M). The network prediction is compared with WNN, PSO_SVM and BP neural network. The results show that the proposed network is superior to the WNN, PSO_SVM and BP neural network. 

#### 4.2.1. Creating the Mother Wavelet Function Library

Because many mother wavelet functions have no specific expression, we cannot work out their derivatives. Therefore, in this work, we only use the following seven mother wavelet functions to bulid a library waveFunciton. Their expressions are as follows:

(1) Haar wavelet function:
  (12) $$\begin{array}{c} \Psi \left(t\right)=\{\begin{array}{cc} 1, & 0\leq t\leq \frac{1}{2}\\ -1, & \frac{1}{2}\leq t\leq 1\\ 0, & \text{other}. \end{array} \end{array}$$
(2) Gaussian wavelet function:
  (13) $$\begin{array}{c} \Psi \left(t\right)=\frac{t}{\sqrt{2\pi }}\exp\left(-\frac{t^{2}}{2}\right). \end{array}$$
(3) Morlet wavelet function:
  (14) $$\begin{array}{c} \Psi \left(t\right)=\cos\left(1.75t\right)\exp\left(-\frac{t^{2}}{2}\right). \end{array}$$
(4) Mexican Hat (Mexihat) wavelet function:
  (15) $$\begin{array}{c} c=\frac{2}{\sqrt{3}}\pi^{-1/4},\\ \Psi \left(t\right)=c\left(1-t^{2}\right)\exp\left(-\frac{t^{2}}{2}\right). \end{array}$$
(5) Shannon wavelet function:
  (16) $$\begin{array}{c} \Psi \left(t\right)=\frac{\sin\pi \left(t-1/2\right)\;-\sin2\pi \left(t-1/2\right)}{\pi \left(t-1/2\right)}. \end{array}$$
(6) Meyer wavelet function (approximate formula):
  (17) $$\begin{array}{c} \Psi \left(t\right)=35t^{4}-84t^{5}+70t^{6}-20t^{7}. \end{array}$$
(7) Wavelet function GGW constructed by the authors:
  (18) $$\begin{array}{c} \Psi \left(t\right)=\sin\left(3t\right)+\sin\left(0.3t\right)+\sin\left(0.03t\right). \end{array}$$

We use the following parameters to initialize the network: the input layer nodes *M* = 6, the hidden layer nodes *n* = 12, the output nodes *N* = 1, and parameter learning rates *lr*_1_ = 0.01 and *lr*_2_ = 0.001, respectively.

We construct wavelet neural network using each wavelet function in wavelet function library and then input training set into network and train the network. The MSEs are as follows (from small to large): Morlet < Mexihat < GGW < Haar < Gaussian < Shannon < Meyer, as shown in Table 2. From Table 2, we can draw the conclusion that running time and MSE of Morlet and Mexihat are slightly different, but the MSE of Gaussian, Shannon, and Meyer is extremely great, completely divorced from reality. The results we got in this paper are consistent with the fact that most papers adopt Mexihat or Morlet as mother wavelet functions to construct wavelet neural network. So, we can use wavelet functions Morlet or Mexihat as mother wavelet function to create MWFWNN network. In this paper, we use the Morlet wavelet function as the basic mother wavelet function to construct MWFWNN.

#### 4.2.2. *Comparing* with WNN and PSO_SVM, BP

Similar to MWFWNN, WNN, BP network, and support vector machine (SVM) can be used to solve the target assessment. As the choice of the support vector machine parameters *c* and *g* has no uniform standard except relying on experience; in this paper, we use particle swarm optimization (PSO) algorithm to optimize the SVM parameters *c* and *g*. Next, we use wavelet neural network, BP neural network, and PSO_SVM to solve threat assessment, and the results are compared with MWFWNN.

The structure of wavelet neural network and BP neural network is 6-12-1 according to the characteristics of the data used. Where the WNN and other parameters are setting as shown in Section 4.2.1. PSO SVM uses LIBSVM toolbox [11] whose default is C-SVR and RBF kernel function, where *C* = 1, *γ* = 1/*n*. By default, PSO local search *c*_1_ = 1.5, the global search *c*_2_ = 1.7, the maximum evolution times maxgen = 200, the maximum population size sizepop = 20, the rate *k* = 0.6 between *V* and *X*  (*k* ∈ [0.1,1.0], *V* = *kX*), SVM parameter *c* ∈ [0.1,100], and the parameter *g* ∈ [0.01,1000].

Threat assessment is predicted by the trained WNN, BP network, and PSO_SVM and their MSE are 4.01 × 10^−3^, 9.39 × 10^−3^ and 4.80 × 10^−3^, all of which are greater than MWFWNN (1.23 × 10^−3^) (as shown in Table 3). For the running time, due to BP neural network calling MATLAB embedded functions, running time is least; wavelet neural networks need to call each mother wavelet function and its derivative, so it is time to consume more; MWFWNN network needs to find the optimal wavelet function from the library, so the running time is more than WNN; the PSO_SVM call PSO algorithm to optimize parameter *c* and *g*, but PSO optimization algorithm is more complex, so it implements most slowly.

**Figure 3:**
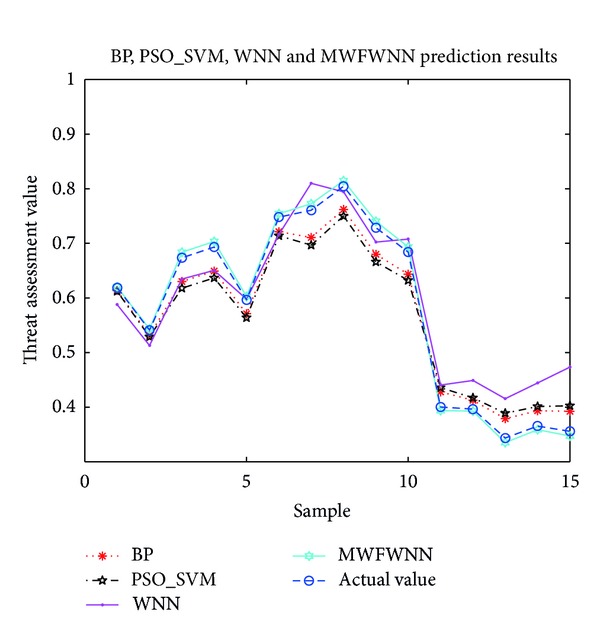
Result of target threat assessment based on WNN, BP, MWFWNN and PSO_SVM.

Prediction error of BP, PSO_SVM, WNN, and MWFWNN is shown in Figure 3. The figure shows that, prediction errors of WNN, BP, and PSO_SVM are simlilar. The error is less than the true value at sample 1–10, while the error was significantly greater than the true value at sample 11–15. MWFWNN network error has similar trend, but the error was significantly less than the WNN, BP network and PSO_SVM, prediction value closer to the expectations.

## 5. Conclusion

Based on requirements for quickly processing information in the modern information war, aiming to the characteristics of threat assessment in data fusion functional model, we adopt MWFWNN to solve the threat assessment under the comprehensive consideration of various factors which influence the threat degree. After constructing wavelet function library with 7 wavelet functions, we get the best performance wavelet function Morlet as mother wavelet function to construct MWFWNN network and its result is compared with the WNN, BP, and PSO_SVM networks. Simulation results show that, the MSE of MWFWNN is 1.23 × 10^−3^, which is far better than WNN (4.01 × 10^−3^), BP network (9.39 × 10^−3^), and PSO_SVM (4.80 × 10^−3^), achieving the desired goal. In our future work, we will further expand the scale of wavelet function library to find more suitable wavelet function to solve the threat assessment and other problems.

## Figures and Tables

**Table 1:** Part of data.

No.	Type	Velocity (m/s)	Heading angle (°)	Inference	Height	Distance (km)	Threat value
1	Large	450	8	Medium	Low	300	0.5843
2	Large	400	3	Strong	High	100	0.5707
3	Large	450	16	Medium	Low	200	0.5333
4	Large	800	4	Strong	High	100	0.6895
5	Large	800	12	Strong	Low	320	0.6896
6	Small	530	6	Strong	Medium	230	0.6056
7	Small	650	8	Strong	Medium	200	0.7425
8	Small	700	12	Strong	Low	320	0.7336
9	Small	750	15	Medium	Very low	400	0.7541
10	Small	640	18	Strong	Medium	280	0.6764
11	Helicopter	90	12	Weak	Very low	320	0.3937
12	Helicopter	110	3	No	Medium	100	0.3927
13	Helicopter	100	9	No	Medium	260	0.3351
14	Helicopter	120	15	No	Low	160	0.3586
15	Helicopter	80	6	Weak	High	180	0.3471

**Table 2:** Predicting results of different mother wavelet function.

Wavelet function	Haar	Gaussian	Morlet	Mexihat	Shannon	Meyer	GGW
MSE	2.10 × 10^−2^	1.03 × 10^54^	1.23 × 10^−3^	1.27 × 10^−3^	1.11 × 10^55^	3.90 × 10^57^	1.60 × 10^−2^
Running time (s)	4.74	4.76	4.85	4.88	4.84	5.24	4.88

**Table 3:** Predicting results of BP, PSO_SVM, MWFWNN, and WNN.

Neural network	BP	PSO_SVM	MWFWNN	WNN
MSE	9.39 × 10^−3^	4.80 × 10^−3^	1.23 × 10^−3^	4.01 × 10^−3^
Running time (s)	1.86	10.6	4.88	5.10
